# Prognostic value of the triglyceride–glucose index for ICU mortality in non-diabetic sepsis: a restricted cubic spline analysis

**DOI:** 10.3389/fendo.2026.1752068

**Published:** 2026-03-09

**Authors:** Ming Min, Dan Gui, Lijun Gong, Qianfei Liu, Yaomei Luo, Yanli Cao, Peijun Liu

**Affiliations:** 1 Department of Respiratory and Critical Care Medicine, The Central Hospital of Enshi Tujia and Miao Autonomous Prefecture, Enshi, China; 2 Department of Endocrinology, The Central Hospital of Enshi Tujia and Miao Autonomous Prefecture, Enshi, China; 3 The Central Hospital of Enshi Tujia and Miao Autonomous Prefecture, Hubei Minzu University, Enshi, China

**Keywords:** external validation, ICU mortality, prognosis, sepsis, survival prediction, TyG index

## Abstract

**Background:**

The triglyceride–glucose (TyG) index has been identified as a metabolic marker associated with adverse outcomes in sepsis, but its prognostic value in non-diabetic septic patients remains unclear.

**Objective:**

To assess the association between TyG levels and ICU mortality in non-diabetic sepsis and validate the findings in an external cohort.

**Methods:**

A retrospective analysis of 2,217 non-diabetic sepsis patients from the MIMIC-IV database was conducted using multivariate logistic regression, threshold effect analysis, restricted cubic spline (RCS) modeling, and subgroup analyses. External validation was performed in an independent ICU cohort of 185 non-diabetic sepsis patients from The Central Hospital of Enshi, stratified by the MIMIC-derived TyG cut-off of 9.163.

**Results:**

ICU mortality in the MIMIC cohort was 12.2%. Compared with Q2, Q4 showed a significantly increased mortality risk (OR = 1.49, 95% CI: 1.12–1.97, P = 0.006). RCS analysis demonstrated a significant U-shaped association with a threshold at TyG = 9.163. Subgroup analyses confirmed consistent trends. In the external validation cohort, mortality was higher in the high-TyG group than in the low-TyG group (29.0% vs. 21.7%), showing a directionally consistent, though non-significant, trend (P = 0.23).

**Conclusion:**

The TyG index is independently associated with ICU mortality in non-diabetic sepsis and exhibits a clear U-shaped pattern. The external validation cohort demonstrated a similar risk trend, supporting the broader applicability of TyG as a simple metabolic marker for risk stratification.

## 1 Introduction

Sepsis represents a potentially fatal disorder marked by compromised organ function stemming from an irregular host reaction to infectious or non-infectious inflammatory processes. Along with systemic inflammatory response syndrome and symptoms at the primary infection site, it is frequently associated with inadequate perfusion of vital organs. Sepsis is a prevalent syndrome in critical care medicine and a leading cause of mortality among intensive care unit (ICU) patients (1, 2). Diabetic individuals exhibit compromised innate and adaptive immune functions, which heighten their susceptibility to infections and the progression of sepsis. With over 900,000 cases annually in the U.S. and an incidence rate of 535 per 100,000 person-years, sepsis remains a major global health challenge (3, 4). Although substantial progress has been made in medical management and standardization of care, sepsis-related mortality remains unacceptably high, ranging from 20% to 36%, with an estimated 270,000 deaths annually in the United States (5–7). Effective management of sepsis is complex, requiring timely diagnosis and intervention to improve outcomes and reduce mortality. Early detection and risk assessment are therefore crucial, with profound implications for clinical treatment and mortality reduction (8).

The triglyceride-glucose (TyG) index, an emerging biomarker for insulin resistance (IR) and metabolic disorders, is derived by calculating the natural logarithm of the product of fasting triglycerides and glucose levels: ln[fasting triglycerides (mg/dL) × fasting glucose (mg/dL)/2]. The TyG index exhibits notable associations with metabolic syndrome, IR, and the likelihood of cardiovascular and cerebrovascular diseases, including coronary artery disease and stroke (9–11). It also correlates with the risk and severity of exacerbations in chronic lung diseases (12). Recent studies suggest that the TyG index may be a valuable predictor of adverse outcomes in critically ill patients, including severe stroke, severe atrial fibrillation, and severe acute exacerbations of chronic obstructive pulmonary disease (COPD), underscoring its potential in the diagnosis and prognosis of sepsis in ICU patients (13–15).

This investigation sought to evaluate the link between the TyG index and ICU mortality in non-diabetic septic patients, providing deeper insights into underlying pathophysiological mechanisms and offering new perspectives for personalized treatment. The findings may enhance clinical decision-making and inform improved management strategies for septic patients.

## 2 Materials and methods

### 2.1 Data source and study design

This retrospective cohort analysis employed the Medical Information Mart for Intensive Care IV (MIMIC-IV) database, which consolidates multidimensional medical data, including demographic information, laboratory results, medication regimens, physiological parameters, disease diagnoses, and prognostic data. The research procedure was sanctioned by the Institutional Review Boards of the Massachusetts Institute of Technology and Beth Israel Deaconess Medical Center (Project ID: 27572725). Owing to the dataset’s de-identified nature and the absence of direct intervention, informed consent was waived. Author Qianfei Liu (Certification ID: 57580527) completed standardized data use training and was responsible for data extraction and quality control. The protocol was reviewed and approved by the Medical Ethics Committee of Enshi Tujia and Miao Autonomous Prefecture Central Hospital (No. 2025-095-01).

### 2.2 Study population screening criteria

The inclusion criteria for this study were: adults aged 18 years or older, an ICU stay of at least 24 hours to ensure data completeness, and a diagnosis of sepsis according to the Sepsis-3 International Consensus criteria. Exclusion criteria included missing key metabolic indicators such as triglycerides, glucose, or TyG index-related parameters; missing data for other core variables exceeding 30%; and a documented history of diabetes or hyperlipidemia, which was applied to minimize potential confounding effects on triglyceride levels and ensure that the TyG index more accurately reflected acute metabolic status rather than chronic lipid disorders.

### 2.3 Data collection and processing

Structured data was extracted using the PostgreSQL-based pgAdmin 4.5 data management platform. Key variables included baseline characteristics (age, sex, race, and BMI), physiological parameters measured within 24 hours of ICU admission, laboratory indicators obtained within 24 hours (fasting glucose and triglycerides), and major clinical comorbidities such as hypertension, heart failure, myocardial infarction, malignancy, chronic kidney disease, and stroke. The TyG index was calculated using the formula: TyG index = ln[fasting TG (mg/dL) × fasting glucose (mg/dL)/2]. Patients were categorized into quartiles (Q1–Q4) based on their TyG index values. Variables with missing rates exceeding 30% were excluded, and remaining missing data were handled using complete-case analysis.

### 2.4 External validation cohort

An external validation cohort was retrospectively constructed using ICU data from the Central Hospital of Enshi Tujia and Miao Autonomous Prefecture. Adult patients (≥18 years) admitted between January 2020 and December 2024 were screened. Sepsis was defined according to the Sepsis-3 criteria. Patients were eligible if they remained in the ICU for ≥24 hours and had fasting triglyceride and fasting glucose measurements obtained within the first 24 hours of admission. Those with documented diabetes, hyperlipidemia, use of lipid-lowering medications, or missing TyG-related variables were excluded. In total, 185 non-diabetic septic patients were included in the validation cohort. The TyG index was calculated using the same formula as in the primary analysis. According to the threshold identified in the MIMIC-IV cohort (TyG = 9.163), patients were categorized into two groups: Low TyG: < 9.163, High TyG: ≥ 9.163. The primary outcome was ICU mortality, defined as death during the index ICU stay.

### 2.5 Statistical analysis

Chi-square tests were used to analyze categorical variables, which were presented as percentages. Skewed continuous variables were expressed as medians with interquartile ranges (IQR), and differences across TyG index quartiles were assessed using the Kruskal-Wallis test. Multivariate logistic regression was conducted to evaluate the association between TyG index levels and ICU mortality across the four groups, with odds ratios (ORs) and 95% confidence intervals (CIs) reported. Stepwise regression models were applied as follows: Model 1 was unadjusted, Model 2 was adjusted for demographic variables including sex, age, race, and BMI, and Model 3 was further adjusted for vital signs and comorbidities. To assess potential nonlinear associations, cubic and penalized spline models were used. Segmented regression analysis was conducted to identify any threshold effects of the TyG index on ICU mortality. Subgroup analyses were also performed to examine the predictive relationship between the TyG index and various patient characteristics. All statistical analyses were carried out using R software, and a two-tailed P-value of less than 0.05 was considered statistically significant.

## 3 Results

### 3.1 Flowchart of participant selection and inclusion criteria

**Figure 1:**
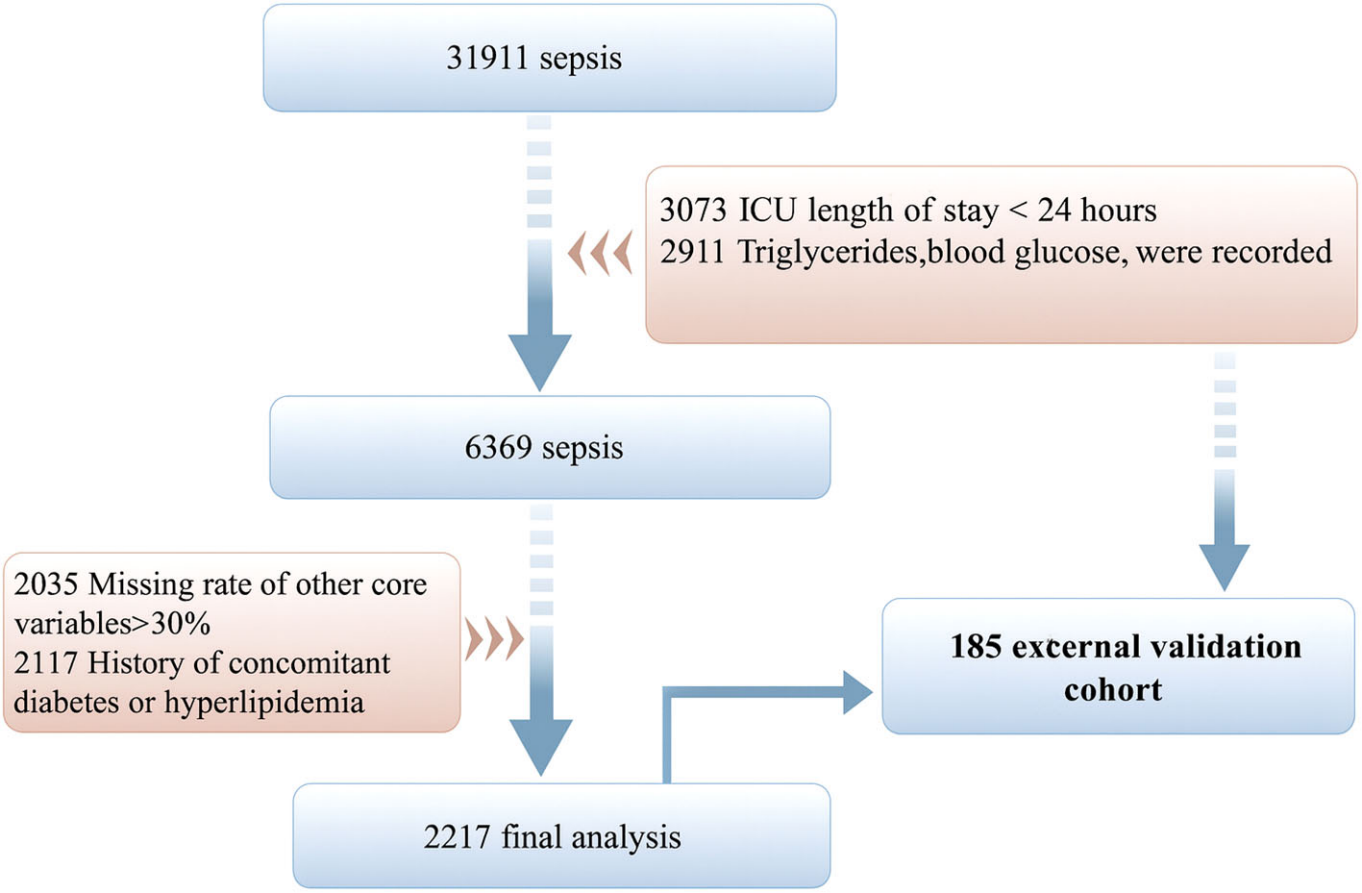
Flow chart of study participants.

After rigorous screening according to predefined eligibility criteria, a total of 2,217 adult ICU patients with sepsis were included in the final analysis. All participants met the diagnostic criteria for sepsis, possessed complete clinical and laboratory data, and had no documented history of diabetes or hyperlipidemia. The detailed selection and exclusion process is illustrated in **Figure 1**, which presents the stepwise progression from the initial dataset to the final study cohort.

### 3.2 Baseline characteristics and comparative analysis

This study included 2,217 participants, distributed as follows: Q1 (n = 554, 24.99%), Q2 (n = 554, 24.99%), Q3 (n = 554, 24.99%), and Q4 (n = 555, 25.03%). Notable statistical variations (*P* < 0.05) emerged among the four groups regarding respiratory rate, heart rate, systolic blood pressure, Sequential Organ Failure Assessment (SOFA) score, Acute Physiology and Chronic Health Evaluation III (APACHE III) score, white blood cell count (WBC), red blood cell count (RBC), platelet count, lactate, total bilirubin, creatinine, heart failure, malignant tumors, stroke, age, and BMI. No significant differences (*P*> 0.05) were noted for diastolic blood pressure, hemoglobin, sex, race, hypertension, myocardial infarction, or chronic kidney disease (**Table 1**).

**Table 1:** Basic characteristics and differential analysis.

Variables	Total (n = 2217)	Q1 (n = 554)	Q2 (n = 554)	Q3 (n = 554)	Q4 (n = 555)	*P*
Heart Rate, M (Q_1_, Q_3_)	95.00 (81.00, 111.00)	91.00 (78.00,106.75)	94.00 (80.25,111.00)	97.50 (84.00,114.00)	98.00 (83.00,113.00)	<.001
Systolic Blood Pressure, M (Q_1_, Q_3_)	117.00 (102.00, 135.75)	115.00 (100.00,131.00)	120.00 (103.00,138.00)	119.00 (102.00,138.00)	116.00 (102.50,132.00)	0.011
Diastolic Blood Pressure, M (Q_1_, Q_3_)	68.00 (58.00, 82.00)	67.00 (57.00,80.00)	70.00 (59.00,83.00)	70.00 (59.00,83.00)	68.00 (58.00,80.50)	0.127
Respiratory Rate, M (Q_1_, Q_3_)	20.00 (16.00, 25.00)	20.00 (16.00,24.00)	20.00 (16.00,25.00)	20.00 (17.00,25.00)	22.00 (18.00,26.00)	<.001
Sofa, M (Q_1_, Q_3_)	7.00 (5.00, 10.00)	7.00 (4.00,10.00)	7.00 (4.00,10.00)	7.00 (4.00,10.00)	8.00 (5.00,11.00)	<.001
Wbc, M (Q_1_, Q_3_)	12.50 (8.20, 17.90)	11.20 (7.88,15.70)	12.70 (8.30,17.72)	12.70 (8.50,18.55)	13.40 (8.60,18.50)	<.001
Rbc, M (Q_1_, Q_3_)	3.60 (2.95, 4.24)	3.46 (2.87,4.10)	3.60 (2.93,4.23)	3.70 (3.06,4.26)	3.65 (2.98,4.30)	0.004
Plateletcount, M (Q_1_, Q_3_)	178.00 (112.00, 254.00)	170.00 (104.00,240.00)	183.00 (117.00,263.00)	188.00 (126.00,264.00)	171.00 (101.75,247.25)	0.007
Lactate, M (Q_1_, Q_3_)	1.90 (1.30, 3.10)	1.80 (1.20,2.88)	1.70 (1.20,2.90)	1.90 (1.30,3.10)	2.20 (1.40,4.00)	<.001
Creatinine, M (Q_1_, Q_3_)	1.10 (0.80, 1.70)	1.00 (0.70,1.60)	1.00 (0.70,1.50)	1.00 (0.72,1.80)	1.30 (0.90,2.10)	<.001
Gender, n(%)						0.401
Male	1338 (60.35)	331 (59.75)	320 (57.76)	346 (62.45)	341 (61.44)	
Female	879 (39.65)	223 (40.25)	234 (42.24)	208 (37.55)	214 (38.56)	
Hypertension, n(%)	668 (30.13)	165 (29.78)	164 (29.60)	167 (30.14)	172 (30.99)	0.960
Heart Failure, n(%)	456 (20.57)	133 (24.01)	130 (23.47)	102 (18.41)	91 (16.40)	0.002
Myocardial Infarction, n(%)	183 (8.25)	38 (6.86)	46 (8.30)	51 (9.21)	48 (8.65)	0.532
Malignant Tumor, n(%)	203 (9.16)	48 (8.66)	69 (12.45)	46 (8.30)	40 (7.21)	0.015
Chronic Kidney Disease, n(%)	235 (10.60)	62 (11.19)	63 (11.37)	58 (10.47)	52 (9.37)	0.694
Stroke, n(%)	137 (6.18)	51 (9.21)	34 (6.14)	33 (5.96)	19 (3.42)	0.001
Age, n(%)						<.001
<60	1195 (53.90)	256 (46.21)	274 (49.46)	311 (56.14)	354 (63.78)	
≥60	1022 (46.10)	298 (53.79)	280 (50.54)	243 (43.86)	201 (36.22)	
BMI, n(%)						<.001
<24	565 (25.48)	204 (36.82)	151 (27.26)	131 (23.65)	79 (14.23)	
<28, ≥24	532 (24.00)	140 (25.27)	155 (27.98)	129 (23.29)	108 (19.46)	
≥28	1120 (50.52)	210 (37.91)	248 (44.77)	294 (53.07)	368 (66.31)	

SOFA, Sequential Organ Failure Assessment; APSIII, Acute Physiology Score III; Wbc, White Blood Cell count; Rbc, Red Blood Cell count; BMI, Body Mass Index.

### 3.3 Multimodel analysis of the link between TYG index and ICU mortality

Multivariate logistic regression analyses were conducted to evaluate the association between TyG quartiles and ICU mortality (**Table 2**). Using Q2 as the reference, the unadjusted model (Model 1) demonstrated a significantly increased mortality risk in Q4 (OR = 1.49, 95% CI: 1.12–1.97, P = 0.006), whereas Q1 and Q3 showed no significant associations. Adjustment for demographic variables in Model 2 yielded similar findings, with the elevated risk in Q4 remaining robust (OR = 1.48, 95% CI: 1.11–1.98, P = 0.008). In the fully adjusted Model 3, which incorporated additional clinical covariates, the association for Q4 persisted though modestly attenuated (OR = 1.36, 95% CI: 1.01–1.83, P = 0.045). Collectively, these results highlight a nonlinear relationship in which markedly elevated TyG levels independently confer an increased risk of ICU mortality among non-diabetic sepsis patients.

**Table 2:** Multimodel strategy analysis of the relationship between TYG index and ICU mortality.

Variables	Model1		Model2		Model3	
	OR (95%CI)	*P*	OR (95%CI)	*P*	OR (95%CI)	*P*
TYG quantile						
Q2	1.00 (Reference)		1.00 (Reference)		1.00 (Reference)	
Q1	1.23 (0.92 ~ 1.64)	0.163	1.24 (0.93 ~ 1.67)	0.143	1.29 (0.96 ~ 1.74)	0.094
Q3	0.90 (0.66 ~ 1.22)	0.488	0.90 (0.66 ~ 1.22)	0.480	0.90 (0.66 ~ 1.23)	0.522
Q4	1.49 (1.12 ~ 1.97)	0.006	1.48 (1.11 ~ 1.98)	0.008	1.36 (1.01 ~ 1.83)	0.045

Model1: Crude.

Model2: Adjust: gender, race, age, BMI.

Model3: Adjust: gender, race, age, BMI, Hypertension, Heart Failure, Myocardial Infarction, Malignant Tumor, Chronic Kidney Disease, Stroke, Heart Rate, Systolic Blood Pressure, Diastolic Blood Pressure, Respiratory Rate.

OR: Odds Ratio, CI: Confidence Interval; BMI, Body Mass Index.

### 3.4 Detection of nonlinear relationships

**Figure 2:**
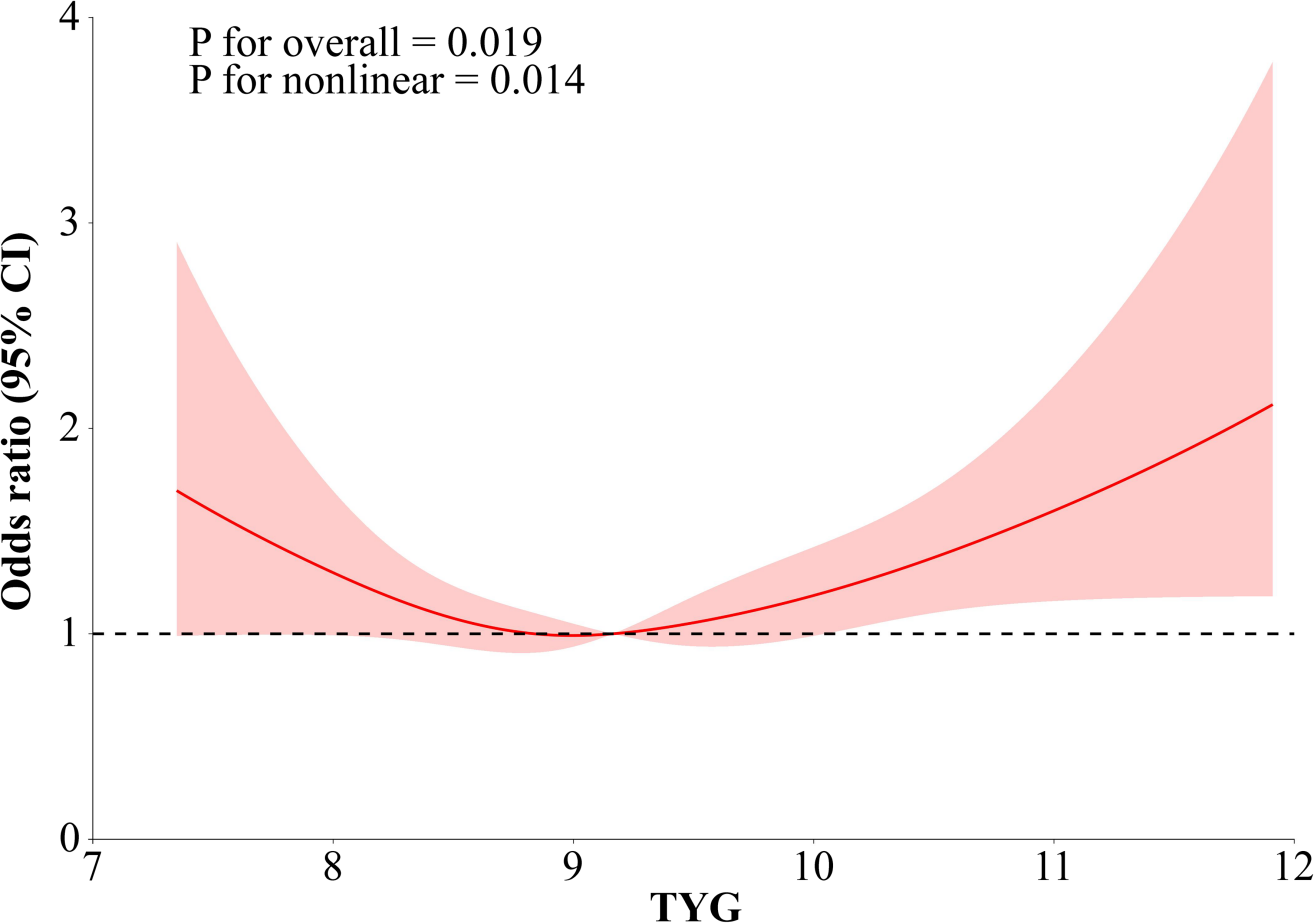
Multivariable-adjusted restricted cubic spline depicting a U-shaped association between the TyG index and ICU mortality.

After adjusting for demographic covariates, including age, race, BMI, and sex, a significant threshold effect was identified between the TyG index and ICU mortality (P for likelihood ratio test = 0.007). As shown in the fitted two-piecewise linear regression model, when the TyG index was below 9.163, an inverse relationship with ICU mortality was observed, suggesting that moderately lower TyG levels might reflect adequate metabolic balance and improved outcomes [OR (95% CI): 0.72 (0.52–1.00)]. In contrast, when the TyG index exceeded 9.163, the association shifted to a positive direction, with markedly increased mortality risk [OR (95% CI): 1.38 (1.10–1.73)]. The RCS analysis demonstrated a clear U-shaped relationship between the TyG index and ICU mortality among non-diabetic septic patients. Both low and high TyG values were associated with increased mortality. In contrast, the lowest risk was observed within the midrange of TyG levels, reflecting a nonlinear, threshold-dependent pattern after full adjustment for confounders. (**Figure 2**; **Supplementary Table 1**).

### 3.5 Subgroup analysis

Subgroup analysis was performed to examine further whether the association between the TyG index (dichotomized at 9.163) and ICU mortality varied across clinical and demographic strata (**Supplementary Table 2**). Consistent trends were observed across all subgroups, with no significant interaction between TyG category and any stratification variable (*P* > 0.05). This stability suggests that the prognostic value of the TyG index is broadly applicable to patients of different ages, sexes, and comorbidity profiles. Moreover, the magnitude of association appeared more pronounced in patients without hypertension or CKD, implying that metabolic dysregulation reflected by an elevated TyG index may exert a stronger independent effect on mortality risk in individuals without overt cardiometabolic disease. Collectively, these results reinforce the robustness and generalizability of the TyG–mortality relationship across heterogeneous clinical settings.

### 3.6 Association between TyG index and ICU mortality

**Figure 3:**
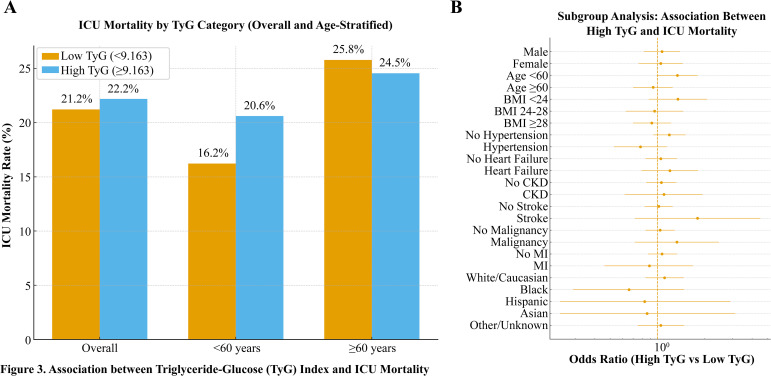
Association between triglyceride–glucose (TyG) index and ICU mortality. **(A)** ICU mortality rate in low (<9.163) and high (≥9.163) TyG groups, stratified by age. **(B)** Subgroup analysis showing adjusted ORs and 95% Cis for the association between high TyG and ICU mortality across demographic and clinical variables.

Based on the identified inflection point (TyG = 9.163), patients were divided into low and high TyG groups to assess the prognostic association with ICU mortality. As shown in **Figure 3A**, mortality was higher in the high TyG group than in the low TyG group (22.2% vs. 21.2%, *P* < 0.05), particularly among patients <60 years (20.6% vs. 16.2%), while no significant difference was observed in those ≥60 years (24.5% vs. 25.8%). Subgroup analyses across demographic and clinical characteristics (**Figure 3B**) confirmed that higher TyG levels were independently linked to increased ICU mortality after adjustment for confounders. No significant interaction between TyG and any subgroup variable was found (*P* > 0.05), indicating a stable relationship across populations. The effect appeared stronger in patients without hypertension or CKD, suggesting a direct metabolic contribution. Collectively, elevated TyG was identified as an independent and consistent prognostic marker for ICU mortality in non-diabetic sepsis patients. Multivariable-adjusted restricted cubic spline depicting a U-shaped association between the TyG index and ICU mortality.

### 3.7 External validation results

A total of 185 non-diabetic septic patients were included in the external validation cohort, with 92 in the low-TyG group (TyG < 9.163) and 93 in the high-TyG group (TyG ≥ 9.163). ICU mortality showed a non-significant but directionally consistent trend across TyG categories. The mortality rate was 21.7% (20/92) in the low-TyG group and 29.0% (27/93) in the high-TyG group, corresponding to a chi-square P value of 0.23. Although not statistically significant, the effect direction mirrored findings from the MIMIC-IV derivation cohort, suggesting a reproducible tendency toward higher mortality with elevated TyG levels (**Table 3**).

**Table 3:** ICU mortality in low versus high TyG groups in the external validation cohort.

Group	n	ICU deaths (n)	ICU mortality (%)	*P*
Low TyG (< 9.163)	92	20	21.7%	
High TyG (≥ 9.163)	93	27	29.0%	
Chi-square test				0.23

## 4 Discussion

The TyG index, functioning as an alternative indicator of IR, demonstrates linkages to adverse outcomes in various populations, including critically ill patients. Recent studies indicate that elevated TyG levels correlate with increased mortality in septic patients, underscoring the role of metabolic dysregulation in prognosis (16, 17). This analysis reveals that the TyG index is independently connected to ICU mortality risk in non-diabetic septic patients, with this association remaining significant after adjusting for demographic and clinical variables. These results suggest the TyG index could function as an effective biomarker for sepsis prognosis, aligning with prior research that highlights IR as a key contributor to poor sepsis outcomes. As a simple and rapid measure of IR, the TyG index reflects disturbances in glucose and lipid metabolism during sepsis (18).

Sepsis triggers IR and lipid metabolism abnormalities, often accompanied by uncontrolled hyperglycemia and glucose fluctuations in the acute phase. The TyG index offers valuable insights for clinical management, indicating the importance of monitoring glucose levels and IR markers in septic patients (19). The TyG index has gained recognition as a promising biomarker linked to various conditions, including metabolic abnormalities, arterial disease, heart-related disorders, and COVID-19 infection (20, 21). Recent investigations have examined its relationship with illness severity and patient outcomes among those suffering from severe medical conditions and infectious pathologies. Given its role in indicating insulin sensitivity impairment and metabolic disruption, the TyG index demonstrates substantial predictive capabilities within intensive care environments and infectious disease management scenarios. For example, elevated TyG levels are linked to acute kidney injury (OR = 1.90) and ICU mortality (HR = 1.81) in patients with aneurysmal subarachnoid hemorrhage. Predictive models using the TyG index (AUC = 0.796) have been developed as online tools to aid clinical decision-making (16). In conditions such as cardiac arrest and cerebral hemorrhage, the TyG index is an independent risk factor for ICU mortality, correlating with disease severity (SOFA score) and mortality (AUC = 0.657–0.828), with blood urea nitrogen partially mediating this relationship (mediation proportion: 12.4%) (22, 23).

Metabolically, the TyG index is linked to a 1.89-fold elevated risk of sepsis in acute pancreatitis patients (OR = 1.891) (24). Elevated TyG levels are also linked to the risk of chronic lung disease, declining lung function, and worsening respiratory symptoms (12). In patients with COPD, every increment in TyG elevates the probability of respiratory failure by 82% (HR = 1.821), with high-TyG groups exhibiting nearly three times the rate of mechanical ventilation usage. These associations are underpinned by IR-driven inflammation, oxidative stress, and disruptions in glucose-lipid metabolism (25). Moreover, sepsis prognosis correlates with the severity of the inflammatory response, with TyG levels positively associated with disease severity scores (26).

In addition to the harmful effects of elevated TyG levels reflecting insulin resistance, increased mortality at lower TyG levels may also have biological explanations. Low TyG values in critically ill patients may indicate reduced metabolic reserve, substrate depletion, and severe catabolism. In sepsis, intense inflammatory and hypermetabolic responses can further lower circulating lipid and glucose levels. Such metabolic exhaustion may reflect poor physiological resilience and contribute to adverse outcomes, partly explaining the observed U-shaped association. This study suggested a U-shaped link between TyG levels and the odds of ICU mortality in sepsis. Threshold analysis using specialized statistical techniques identified a significant threshold effect after adjusting for age, race, BMI, and sex. The curve initially decreased and subsequently increased, with an inflection point at a TyG value of approximately 9.163, at which the odds ratio for ICU mortality equaled 1. Variations beyond this reference point, in both upward and downward directions, correlated with gradually rising probabilities of death. These findings may facilitate earlier and more effective interventions for septic patients, potentially reducing mortality rates.

In the subgroup analysis of 2,217 septic patients, dichotomizing the TyG index at the 9.163 threshold confirmed consistent associations between TyG levels and ICU mortality risk across diverse subgroups, encompassing age, sex, race, and comorbidities. No notable interactions were detected between the TyG index and these stratification variables, highlighting its robustness as a prognostic biomarker for sepsis. The TyG index, as an easily obtainable metric, provides useful support in the prognostic assessment and management of septic patients (27, 28). Additional investigations are essential to explore the mechanistic role of the TyG index in sepsis and conduct randomized controlled trials to further validate its prognostic utility (29). In the external validation cohort, the high-TyG group demonstrated a numerically higher ICU mortality compared with the low-TyG group (29.0% vs. 21.7%). However, this difference did not reach statistical significance (P = 0.23). This lack of statistical significance is likely attributable to the relatively small sample size and limited number of outcome events in the validation cohort, which may have reduced statistical power. Importantly, the direction and magnitude of effect were consistent with those observed in the MIMIC-IV derivation cohort, supporting the reproducibility of the association. These findings provide supportive but preliminary external validation and highlight the need for larger, multicenter studies to confirm further the robustness and generalizability of the TyG–mortality relationship. Collectively, these results offer preliminary external support for the generalizability of the TyG–mortality association and emphasize the necessity of larger, multicenter investigations to confirm these findings further.

Furthermore, studies that incorporate additional confounders, such as genetic background, lifestyle, and environmental factors, could provide deeper insights into how the TyG index affects sepsis-related clinical outcomes. Despite its valuable insights, this study has several limitations. First, the MIMIC-IV database is derived from a U.S. population, which may limit the generalizability of our findings to other ethnicities and geographical regions. Second, given the observational design of this study, causal inferences cannot be established. Third, potential biases, including information bias and missing data, might have influenced the accuracy and robustness of the results. Finally, residual confounding cannot be fully excluded, as not all potential covariates were available or adjusted for.

## 5 Conclusion

This study confirmed a significant U-shaped link between the TyG index and ICU mortality risk in individuals with non-diabetic sepsis, with a clinically relevant risk threshold identified. As a simple and accessible metabolic marker, the TyG index holds considerable potential for prognostic assessment and personalized management in sepsis, offering a novel tool and intervention target for clinical practice.

## Data Availability

The original contributions presented in the study are included in the article/**Supplementary Material**. Further inquiries can be directed to the corresponding author.
