# Supplementary material for: Prognostic value of the triglyceride–glucose index for ICU mortality in non-diabetic sepsis: a restricted cubic spline analysis
Source: Front Endocrinol (Lausanne). 2026 Mar 9;17:1752068. doi: 10.3389/fendo.2026.1752068 (PMC13006306; doi:10.3389/fendo.2026.1752068)
Supplement: Supplementary file 1 [file Table1.docx]

**Supplementary Table 1: Detection of Nonlinear Relationships**

| Outcome | Effect OR (95%CI) | *P* |
| --- | --- | --- |
|  |  |  |
| Model 1 Fitting model by standard linear regression | 1.07 (0.94 - 1.21) | 0.316 |
| Model 2 Fitting model by two-piecewise linear regression |  |  |
| Inflection point | 9.16 |  |
| <9.16 | 0.72 (0.52 - 1.00) | 0.050 |
| ≥9.16 | 1.38 (1.10 - 1.73) | 0.005 |
| P for likelihood test |  | 0.007 |
| Abbreviations: OR: Odds Ratio, CI: Confidence Interval |  |  |
