# Supplementary material for: Prognostic value of the triglyceride–glucose index for ICU mortality in non-diabetic sepsis: a restricted cubic spline analysis
Source: Front Endocrinol (Lausanne). 2026 Mar 9;17:1752068. doi: 10.3389/fendo.2026.1752068 (PMC13006306; doi:10.3389/fendo.2026.1752068)
Supplement: Supplementary file 2 [file Table2.docx]

### Supplementary Table 2: Subgroup Analysis

| Variables | n (%) | Lower_ TYG | Higher_ TYG | OR (95%CI) | *P* | P for interaction |
| --- | --- | --- | --- | --- | --- | --- |
|  |  |  |  |  |  |  |
| Gender |  |  |  |  |  | 0.942 |
| Male | 1338 (60.35) | 136/651 | 151/687 | 1.07 (0.82 ~ 1.39) | 0.628 |  |
| Female | 879 (39.65) | 99/457 | 95/422 | 1.05 (0.76 ~ 1.45) | 0.762 |  |
| Race |  |  |  |  |  | 0.801 |
| White or Caucasian | 1260 (56.83) | 122/656 | 122/604 | 1.11 (0.84 ~ 1.47) | 0.473 |  |
| Black | 178 (8.03) | 17/89 | 12/89 | 0.66 (0.29 ~ 1.48) | 0.312 |  |
| Hispanic | 67 (3.02) | 6/31 | 6/36 | 0.83 (0.24 ~ 2.91) | 0.775 |  |
| Asian | 53 (2.39) | 6/25 | 6/28 | 0.86 (0.24 ~ 3.13) | 0.823 |  |
| Other or Unknown | 659 (29.72) | 84/307 | 100/352 | 1.05 (0.75 ~ 1.48) | 0.765 |  |
| Hypertension |  |  |  |  |  | 0.065 |
| No | 1549 (69.87) | 167/779 | 189/770 | 1.19 (0.94 ~ 1.51) | 0.146 |  |
| Yes | 668 (30.13) | 68/329 | 57/339 | 0.78 (0.53 ~ 1.15) | 0.202 |  |
| Heart Failure |  |  |  |  |  | 0.606 |
| No | 1761 (79.43) | 168/845 | 190/916 | 1.05 (0.84 ~ 1.33) | 0.654 |  |
| Yes | 456 (20.57) | 67/263 | 56/193 | 1.20 (0.79 ~ 1.81) | 0.400 |  |
| Myocardial Infarction |  |  |  |  |  | 0.577 |
| No | 2034 (91.75) | 210/1024 | 219/1010 | 1.07 (0.87 ~ 1.33) | 0.516 |  |
| Yes | 183 (8.25) | 25/84 | 27/99 | 0.89 (0.46 ~ 1.68) | 0.710 |  |
| Malignant Tumor |  |  |  |  |  | 0.472 |
| No | 2014 (90.84) | 205/991 | 219/1023 | 1.04 (0.84 ~ 1.29) | 0.691 |  |
| Yes | 203 (9.16) | 30/117 | 27/86 | 1.33 (0.72 ~ 2.46) | 0.368 |  |
| Chronic Kidney Disease |  |  |  |  |  | 0.911 |
| No | 1982 (89.40) | 201/983 | 214/999 | 1.06 (0.85 ~ 1.32) | 0.594 |  |
| Yes | 235 (10.60) | 34/125 | 32/110 | 1.10 (0.62 ~ 1.94) | 0.748 |  |
| Stroke |  |  |  |  |  | 0.235 |
| No | 2080 (93.82) | 224/1023 | 235/1057 | 1.02 (0.83 ~ 1.25) | 0.853 |  |
| Yes | 137 (6.18) | 11/85 | 11/52 | 1.80 (0.72 ~ 4.52) | 0.208 |  |
| Age |  |  |  |  |  | 0.089 |
| <60 | 1195 (53.90) | 86/530 | 137/665 | 1.34 (0.99 ~ 1.80) | 0.054 |  |
| ≥60 | 1022 (46.10) | 149/578 | 109/444 | 0.94 (0.70 ~ 1.25) | 0.654 |  |
| BMI |  |  |  |  |  | 0.331 |
| <24 | 565 (25.48) | 61/355 | 46/210 | 1.35 (0.88 ~ 2.07) | 0.167 |  |
| <28, ≥24 | 532 (24.00) | 63/295 | 49/237 | 0.96 (0.63 ~ 1.46) | 0.848 |  |
| ≥28 | 1120 (50.52) | 111/458 | 151/662 | 0.92 (0.70 ~ 1.22) | 0.579 |  |
| Abbreviations: TYG,Triglyceride-Glucose Index;BMI,Body Mass Index; OR: Odds Ratio, CI: Confidence Interval | | | | | | |
